# 3D + time blood flow mapping using SPIM-microPIV in the developing zebrafish heart

**DOI:** 10.1364/BOE.9.002418

**Published:** 2018-04-27

**Authors:** Vytautas Zickus, Jonathan M. Taylor

**Affiliations:** School of Physics and Astronomy, University of Glasgow, Glasgow, G12 8QQ, UK

**Keywords:** (110.4153) Motion estimation and optical flow, (120.7250) Velocimetry, (170.2520) Fluorescence microscopy, (170.3880) Medical and biological imaging, (180.6900) Three-dimensional microscopy

## Abstract

We present SPIM-μPIV as a flow imaging system, capable of measuring *in vivo* flow information with 3D micron-scale resolution. Our system was validated using a phantom experiment consisting of a flow of beads in a 50 μm diameter FEP tube. Then, with the help of optical gating techniques, we obtained 3D + time flow fields throughout the full heartbeat in a ∼3 day old zebrafish larva using fluorescent red blood cells as tracer particles. From this we were able to recover 3D flow fields at 31 separate phases in the heartbeat. From our measurements of this specimen, we found the net pumped blood volume through the atrium to be 0.239 nL per beat. SPIM-μPIV enables high quality *in vivo* measurements of flow fields that will be valuable for studies of heart function and fluid-structure interaction in a range of small-animal models.

## 1. Introduction

*In vivo* blood flow mapping within the heart in 3D is of significant interest in cardiac development studies [2], since 3D blood flow information (particularly near the walls) is essential for accurate wall shear stress (WSS) estimation and fluid-structure interaction (FSI) modelling. However, many issues make these measurements challenging, including depth sectioning, adequate tracer seeding density and contrast, background clutter and large variations in velocity with position and time. To avoid the challenges of making direct measurements, some groups have opted for estimation of WSS by using computational fluid dynamics (CFD) modelling with wall motion information [3], but experimental flow measurements have a vital role to play in validation of models, as as well offering a much more direct means to make reliable flow measurements directly *in situ*.

Heart *geometry* has previously been studied *in vivo* in small animal models using an array of imaging systems such as MRI and CT [4], optical coherence tomography (OCT) [5], and fluorescent imaging modalities such as confocal [6] and selective plane illumination microscopy (SPIM) [7]. However, in contrast, only a few works have attempted to quantitatively reconstruct 3D *flow* in the heart from experimental measurements. The zebrafish is one particularly well-researched animal model that is well-matched to fluorescence microscopy applications due to its optical transparency at a young age. The relative simplicity of the two-chamber zebrafish heart makes it an attractive target for 3D mathematical modelling of flow and structure [8], however, previous flow *imaging* work has mostly been limited to measuring blood flow from images representing one single 2D projection [9–11].

Lu et al. [12] used epifluorescence microscopy with Defocusing Digital Particle Image Velocimetry (DDPIV) and microinjected beads in a 32 hours post fertilization (hpf) zebrafish, but only measured instantaneous velocity at six three-dimensional locations in the atrium during diastole, lacking the resolution to reveal the whole flow field. Chan and Liebling [13] used brightfield (BF) microscopy to image the zebrafish heart at three different orientations (at 60 hpf) and then used optical flow analysis in conjunction with a divergence-free fitting method to enable them to smooth out the substantial errors visible in their raw BF flow measurements, and reconstruct a full 3D flow field. However, flow measurements based around full-volume illumination (including brightfield imaging) will typically suffer from velocity underestimation. This effect is called Depth Of Correlation (DOC) [14], an experimental parameter characterising the depth over which out-of-focus particles contribute to the flow estimation, and hence defining the effective *z*-resolution for velocimetry. In the present paper we will in fact show evidence that the problem can be even more severe than a degradation of effective resolution, and can lead to more extreme biases associated with brightfield imagery of flows with spatially-varying velocity gradients.

To tackle the lack of simultaneous quantitative heart geometry and flow information in 3D, and to alleviate DOC effects, we have combined three separate techniques:

- Micro-particle image velocimetry (μPIV), a non invasive flow measurement technique derived from the macro-scale PIV techniques originally developed in the field of aerospace engineering [15, 16].
- Selective plane illumination microscopy (SPIM), a variant of fluorescence light sheet microscopy, allows us to isolate individual planes within the heart for imaging, so that we have true 3D imaging resolution instead of imaging 2D projections of the heart chambers. Thus we can overcome the well-known inherent flow underestimation issues associated with full volume illumination μPIV measurements [14, 18].
- Optical gating techniques [7, 19, 20] enable us to identify the heart phase associated with each raw PIV frame pair. This allows us to extend the concept of *correlation averaging* [21] to the non-stationary periodic flow environment of the circulatory system, combining noisy partial information from multiple heartbeats in a statistically robust manner.

When used together, these enable us to achieve high-fidelity depth-resolved flow and structure reconstruction. We will show that this approach can be used to map reliable 3D blood flow fields in the beating zebrafish heart and blood vessels throughout the heartbeat.

## 2. Method

### 2.1. Imaging and PIV analysis

**Fig. 1:**
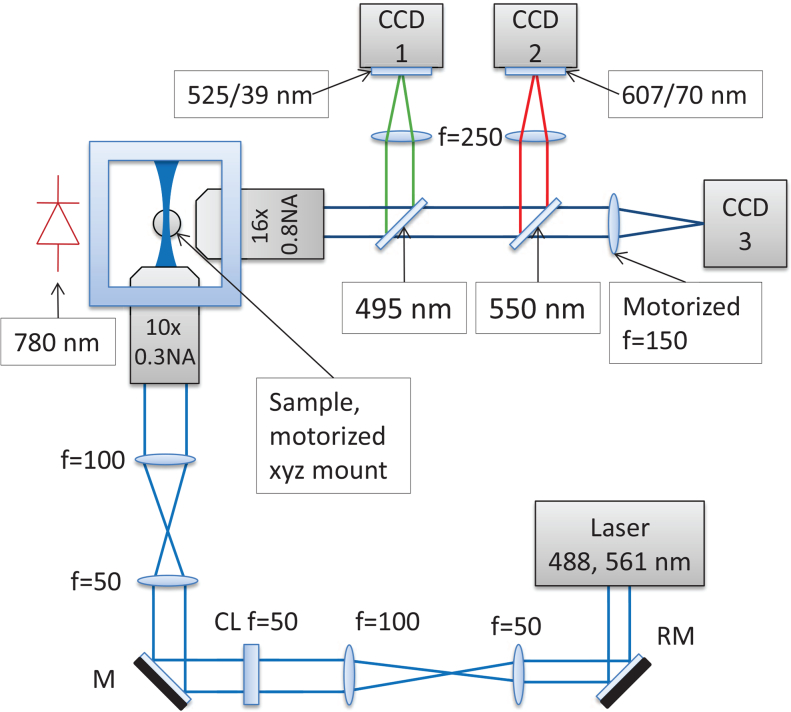
Our SPIM microscope is based around a *Nikon* 10× 0.3NA air objective in the launch path and a *Nikon* 16× 0.8NA water dipping objective in the imaging path. A *Coherent Obis* 488 nm laser (15 mW) was used for the tube flow experiments, and an *Omicron Versalase* multiwavelength laser system was used at 561 nm wavelength (30 mW) for zebrafish RBC imaging. PIV frame pairs were recorded using *QImaging QIClick* CCD cameras (CCD1, CCD2), and brightfield images for heart synchronization were recorded using an *Allied Vision GS650* CCD camera (CCD3). *Chroma* T495lpxr-UF2 and T550lpxr-UF2 dichroics were used for the green and red channels respectively. Further, *Thorlabs* MF525-39 and *Semrock* FF01-607/70-25 filters were used at CCD 1 and 2 respectively. Shadow effects on the illumination arm were minimized using a resonant mirror (RM) after [22], which is essential to prevent shadow artefacts that would bias the PIV analysis.

For fluorescence imaging we use a SPIM microscope based on [23], which, just like conventional PIV imaging systems, uses a cylindrical lens to form a light sheet. The sheet is then focused by a microscope objective to a thickness of ∼2 μm at Full Width at Half Maximum (FWHM), giving excellent depth sectioning, while the orthogonal geometry minimizes exposure of the live sample to laser light. The optical design is shown in Fig. 1

. As is usual in light sheet microscopy, we acquire a fluorescence *z* stack by translating the sample through the light sheet; in order to maintain a fixed focus for the brightfield channel in spite of this translation, we dynamically refocus the brightfield channel using a motor-driven tube lens [19] in order to maintain a consistent heartbeat synchronization reference.

This optical setup mirrors standard macroscopic PIV flow measurements, which use a pulsed, thin sheet of light to illuminate fluid uniformly *seeded* with particles (typical size in the range of 1–10 μm diameter), referred to as tracers, which can be used as light scatterers or sources of fluorescence emission. The measured motion of these particles serves as a proxy for the flow of the host fluid itself. Images of the flowing particles are taken in pairs and the flow is determined by analyzing the tracer motion between frame pairs. A small time difference *dt* between the two frames is chosen to allow sufficient temporal resolution for resolving the underlying time-dependent changes in the flow, and the laser pulse length is carefully selected to maximize the signal while avoiding motion blur of the particles.

In order to test SPIM as a μPIV imaging modality, as well as to compare results with BF μPIV, we imaged flow of water in a Fluorinated Ethylene Propylene (FEP) tube (*ZEUS*, Lot Nr: 201004255-1, 0.0019-in. (1 in. = 2.54 cm) inner diameter, 0.0059-in. outer diameter), seeded with fluorescent polystyrene 1.04 μm diameter beads (*Bangs Laboratories*, Catalog Code: FC04F).

#### 2.1.1. Preparation

The beads were prepared by washing them in purified de-ionized (DI) water, and then sonicating for 10 minutes in an ultrasonic bath to minimize aggregation. Finally, the beads were resuspended in DI water, with a volume ratio of 1:6.25 bead solution (1% solids) to DI water. The FEP tube was washed in acetone and then in DI water before attaching it to a microsyringe pump. The tube was purged with the fluid to ensure an air-free system (to minimize compliance), and the flow was then allowed to settle to a steady state at a fixed flow rate. The mounting of the tube, and the orientation of the cameras were adjusted in such a way that the image of the tube would appear as horizontal as practically possible, so that the plane of the flow was aligned with the plane of the laser sheet and the focal plane of the objective. The main parameters used for imaging and velocimetry are summarized in Table 1

**Table 1:** Main experimental and PIV analysis parameters summarized for both the phantom validation experiment and the zebrafish heart. Effective single pixel size was 0.3225 μm (at 20× magnification). The number of frame pairs is approximate, as it varied slightly from one plane to another due to our acquisition code. The variation was greater for the heart data, however, phase bins had around 50 frame pairs. (*) days post fertilization.

Parameter	Beads in 50 μm ø FEP, flow rate 0.5 \ 1.0 μl/min	fRBCs in the ∼3 dpf* Zebrafish heart
Laser pulse length, ms	0.02 \ 0.01	1
Inter-pulse time, ms	0.3 \ 0.5	1.5
*z*-step, μm	2	8
Camera pixel binning	1 × 1	2 × 2
Frame pairs per *z*-step	∼250 \ 150	∼ 50
Small IW (LxH), px	32 × 12	24 × 24
Big IW (LxH), px	128 × 16	72 × 48
Peak-to-peak threshold	1.10	1.07
Mask around peak, px	7 × 7	7 × 7

. For FEP tube experiments, both the 488 nm laser and the red LED were pulsed simultaneously. The flow rate used for beads in water was 0.5 μl/min and 1.0 μl/min. These flow rates were sufficient to prevent bead deposition in the system and the required pressure did not cause leakage.

#### 2.1.2. μPIV analysis

Once the paired images of the flow are acquired, they are divided into a grid of smaller regions, called interrogation windows (IWs). Broadly speaking, it is the size of IWs that determines the spatial resolution of the calculated flow field. Typically, IWs will be several tens of pixels across, although correlation averaging techniques [24] allow a trade-off between IW size and number of frame pairs acquired. Corresponding IWs from each of the two frames in a pair are then cross-correlated. The peak of the cross-correlation matrix gives the statistically most probable shift of the IW image in time *dt*, which is further fitted to a sub-pixel accuracy [16]. We briefly note that the reliability the estimated motion between two frames depends mostly on the number of particles per IW (the rule of thumb in the PIV literature is that an IW should have on average 10 particles for reliable estimation [16, p.350]). The use of a larger IW in the second image of a frame pair ensures that the same particles present in the first image are still present within the IW in the second image (as long as *dt* is chosen appropriately).

**Fig. 2:**
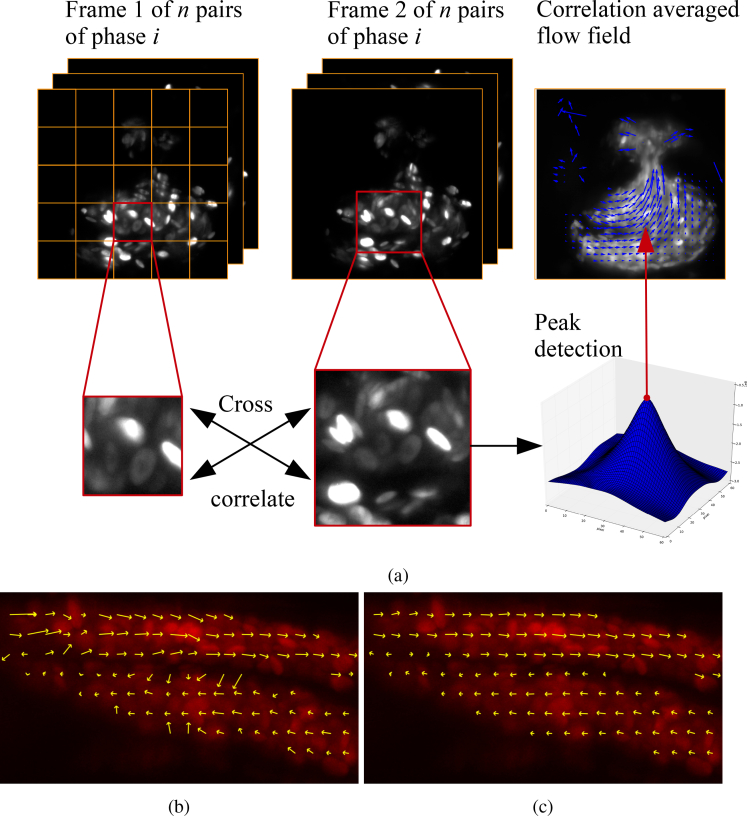
The PIV algorithm, and comparison between two shift estimation methods. (a) Sub-regions of the frame pairs are cross-correlated in time, yielding a matrix of the cross-correlation result, the peak of which then indicates the most likely motion of particle image between the two frames. Peak detection is then followed by subpixel interpolation of the peak and certain criteria tests probing the validity of the measurement, details in [25]. In case of correlation-averaging, each result IW pair matching from the same phase is averaged before peak fitting is executed. (b) shows the standard analysis based on *direct cross correlation*, while (c) illustrates our substitution of the *sum of absolute differences* metric in the cross-correlation stage of the analysis. Notice that the calculated flow in (b) is biased towards high intensities and produces vectors which appear to be pointing inwards, towards the center of the blood vessel. The same parameters were used in the PIV algorithm for both analyses.

We adopted this standard PIV analysis framework for our μPIV analysis of brightfield and fluorescence image data, and modified the OpenPIV python code [25] to enable the use of different sized IWs and to support correlation averaging for robust results on sparsely seeded raw data - see Fig. 2

for an example of correlation averaging process when imaging the heart. By correlation averaging 250 frame pairs with a nominal particle density per small IW of 0.2, the estimated number of tracers should be ∼50 (the reason for having a low seeding density was to mimic potential *in vivo* experiments with microinjected beads - the rationale is that a low seeding density would minimally perturb the natural state of blood flow). The assumption of a steady-state flow meant that correlation averaging could be achieved simply by averaging all the cross-correlation results for each IW in a given *z* plane before estimating the peak position. Our frame pairs were grouped by *z* plane, and the IW dimensions were chosen to ensure that the image in the small IW from the first frame is contained in the larger IW in the second frame. Further, a 50% IW overlap was chosen in an attempt to bring out small scale variations in the flow. We identified unreliable vectors as follows. Once the peak position was found, the area around it extending 3 matrix elements in each direction from the peak, giving a 7 × 7 submatrix, was masked and the next highest value in the unmasked matrix was evaluated to test if it passes a 1.10 ratio threshold, between the peak value and the next highest value in the correlation matrix (referred to as *peak-to-peak* threshold in OpenPIV). Most of the anomalous-looking vectors that were not eliminated by this thresholding test were removed by discarding velocities which had a negative horizontal component, and by discarding the values outside the nominal tube radius (discussed in more detail in the Results section). The python scripts are available in the data repository, as well as on github (https://github.com/vzickus/BOE_323258).

Outlier vectors that escaped the first peak-to-peak threshold test generally lay near the edges of tube boundaries, where very few beads appeared in the images, meaning that, even with correlation averaging, there was insufficient flow seeding. We emphasize the fact that no other pre/post-processing was carried out on the raw data or the calculated velocity fields.

We note that instead of using the traditional cross-correlation function, where image matching is performed by maximizing an intensity product as a function of image shift, we opted for the sum-of-absolute-differences (SAD) measure. Empirically we found this to perform better, especially in the case of non-pointlike tracers (such as fRBCs), or when imaging in BF. Figure 2(b,c) gives an illustration of how direct cross-correlation fails in the common biological scenario of non-uniform intensity distributions. Because the cross-correlation is biased to regions of higher intensity, flow vectors at the edge of a feature are incorrectly rotated in towards the center of the feature. Subtler effects are also seen where a single RBC of unusually high fluorescence intensity can bias the flow in its vicinity. We have not observed either of these effects when using the SAD measure, which does not have the same intensity bias.

### 2.2. Out-of-plane motion tolerance

We investigated the amount of out-of-plane motion (OOPM) that still allows the in-plane motion components to be measured to within an acceptable level of error, when using our SPIM-μPIV system in conjunction with correlation averaging. It is known that OOPM reduces the amplitude of the true peak in the correlation matrix [17, p.176], thus increasing the random error on the calculated in-plane motion. This occurs because some tracers will be lost/gained between the two frames in the PIV frame pair. However, this impact of this source of random error can be reduced through the use of correlation averaging to improve the noise characteristics of the cross-correlation matrix [33].

To verify the level of OOPM that can be tolerated in a situation comparable to our zebrafish imaging scenario, we synthesized a known amount of OOPM by scanning a sample of sparse fluorescent polystyrene 7.32 μm diameter beads (*Bangs Laboratories*, Catalog Code: FS06F) fixed in 1% agarose in a 1.3/1.6 mm inner/outer diameter FEP tube (*Adtech*, part number FT1.3×1.6) through the light sheet. The bead size was chosen to be similar to the size of RBCs, to make the experimental scenario as similar as possible to real blood-flow images.

Frame pairs were selected at a chosen *z* spacing of Δ*z* (simulating out-of-plane motion of distance Δ*z*), and the second frame in each pair was synthetically shifted by 24 pixels (simulating in-plane motion of approximately 7 μm or one tracer diameter). An independent set 8–10 such frame pairs (depending on the value of Δ*z*) were analyzed using one IW covering the whole field of view, and the results combined using correlation averaging to recover an in-plane flow vector, with approximately 200 particle images contributing to each calculated flow vector (estimated using ImageJ’s watershed processing and particle analyzer [31] on a maximum intensity projection of all the images used for the analysis for a single Δ*z*).

By comparing the calculated flow vectors to the known synthetic shift, we can estimate the accuracy of the calculated in-plane motion in the presence of OOPM.

### 2.3. μPIV in the zebrafish heart

To demonstrate the capabilities of SPIM-μPIV *in vivo*, we imaged the larval zebrafish heart. The larvae we imaged were expressing transgenic fluorescent labelling of both the red blood cells (gata:DsRed) and the endothelium lining the blood vessels and heart (flk1:GFP). The larvae were anaesthetized with Tricaine Methanesulfonate (168 mg/L solution) prior to mounting them in an FEP tube with 1% agarose gel to inhibit residual movement. Fluorescence imaging parameters are summarized in Table 1.

**Fig. 3:**
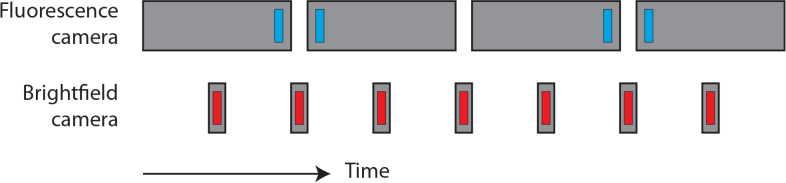
Timing of fluorescence and brightfield camera (BF) exposures (grey boxes), and their associated pulsed illumination (blue and red boxes). By laser-illuminating the sample only for short intervals at the end and beginning of successive fluorescence camera exposures, we were able to sample the flow with better temporal resolution than that implied by the maximum framerate of the camera. Furthermore, the BF images used for heart synchronization may be contaminated by stray laser/fluorescence light leaking through the BF filters; this cross-talk can be avoided by sequencing the BF camera exposures to avoid the laser pulses, as shown here.

We note that pixel binning and a relatively large *z*-step size were chosen to keep the raw data to a manageable size (9 GB fluorescence data and 12.4 GB accompanying brightfield video for heart phase analysis). The acquisition times of the fluorescence and brightfield cameras were sequenced in such a way as to prevent BF channel contamination with laser/fluorescence light (see Fig. 3

). To speed up the analysis, we excluded IWs with a mean intensity count < 10^5^ (i.e. IWs that are effectively black due to not overlapping with the blood pool of the heart). A peak-to-peak threshold of 1.07 was applied in conjunction with a square mask of radius 3, and we emphasize that no additional pre-processing or filtering was carried out on the raw data or the resultant flow fields.

Although the depth-sectioning ability of the SPIM microscope is what enables us to perform *z*-resolved μPIV, this reduces the number of blood cells visible in any individual image (relative to brightfield images), severely compromising the quality of the flow fields that can be reconstructed. As noted earlier, correlation averaging can overcome this by combining information from multiple time-sequential frame pairs – but in the circulatory system the flow is non-stationary, albeit periodic. We therefore use simultaneously-recorded brightfield video for phase analysis. Then, by cross-referencing image timestamps between the brightfield and fluorescence channels, we were able to assign each fluorescence image a heartbeat phase between 0 and 2*π*. The μPIV frame pairs were then grouped according to phase (using bins of size ∼0.209 radians in order to use a reasonable number of frame pairs for correlation averaging, while ensuring that the flow features did not vary too significantly over this time window), and a correlation-averaged μPIV analysis was performed within each phase bin.

## 3. Results and discussion

### 3.1. Phantom experiment results

**Fig. 4:**
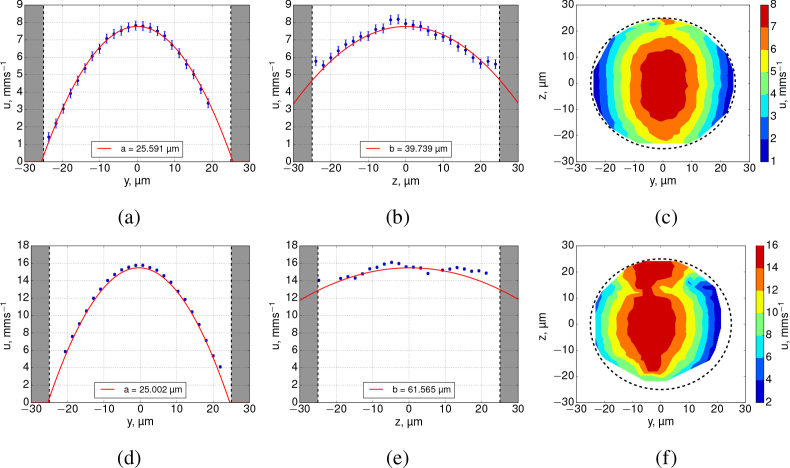
PIV analysis results of the FEP tube experiment data acquired using BF channel. Top row: 0.5 μl/min nominal flow rate beads; bottom row: 1 μl/min beads. Profiles are shown at the location of the peak flow. Shaded area represents tube walls. The profiles of the *u*-components of velocity in *xy* (a,d) have very similar shapes for both flow rate values (semi-minor axis ranges ≈25.0 – 25.6 μm). However, the *xz* plane (b, e) data reveal that the experimentally recovered flow profile stretches with increasing flow rate (semi-major axis increasing from ≈39.8 to 61.6 μm). The contours (c,f) of the *x*-velocity magnitudes in the *yz* plane illustrate the apparent elongation of the parabolic flow profile along the *z*-axis when measured using BF (f) which does not occur in fluorescence, see Fig. 5. Dashes show a diameter of 50 μm.

The results in this section, imaging and analyzing fully-developed Poisseuille flow in a transparent tube (Dataset 1 [26]), serve to validate our SPIM-μPIV system. Figure 4

shows cross-sections along the *xy* and *xz* planes, plotting the *u*-component (which is parallel to the horizontal *x*-axis by convention) of the raw velocity data obtained in BF in a 50 μm diameter tube containing flowing water seeded with 1.04 μm beads.

We performed a single parametric fit to each of our complete 3D experimental datasets, using a model based on Poisseuille flow but allowing for the possibility of a different parabolic profile in the *y* and *z* directions (explained further below). Our model equation is:

(1) $$V(x,y,z)=V_{\text{max}}\left(1-\left(\frac{{\left(y-y_{c}\right)}^{2}}{a^{2}}+\frac{{\left(z-z_{c}\right)}^{2}}{b^{2}}\right)\right),$$

where *V*_max_ is the peak velocity of the flow, *y_c_* and *z_c_* are the locations of the centre of the flow axis in the *y* and *z* planes (*x* centre is assumed to be the centre of the image along its length), *a* and *b* are the semi-minor and major axes of the flow profile. The model fitted to our data also allowed for the possibility of a tilt of the tube in *yz* and *xz* planes, however, it was found not to exceed 2° in *yz*, and the tilt in the *xy*-plane was even more negligible as the camera’s sensor was aligned with the tilt of the tube. We used a two pass fit approach, the initial pass gave an estimate of the parameters which were used to transform the raw data coordinates to tube coordinates, and the transformed points outside of the nominal tube radius were discarded.

A correct 3D reconstruction of the flow in a circularly-symmetric tube will have *a* = *b*, the equation for an ideal paraboloid, but if our experimentally recovered flow suffers from issues such as DOC in the *z* direction then we will find *a* ≠ *b*. Thus we treat *a* and *b* as independent parameters, to serve as a measure of the quality of our flow reconstruction. We found that the semi-minor axes, *a*, were consistent with the measured tube radius of 26.450 ± 0.645 μm, deviating only by 3.25% for the 0.5 μl/min and 5.47% for the 1 μl/min BF datasets. Increasing the flow rate, did not significantly change the parameters of the profile in the *xy*-plane (Fig. 4(a,b)), however, it did drastically increase the semi-major axis, *b*, in the *xz*-planes (Fig. 4(d)–(f)). The ratio between *b* and *a* for 0.5 and 1.0 μl/min flow rate experiments were ≈ 1.55 and ≈ 2.46 respectively, indicating that the recovered flow profile is incorrect.

**Fig. 5:**
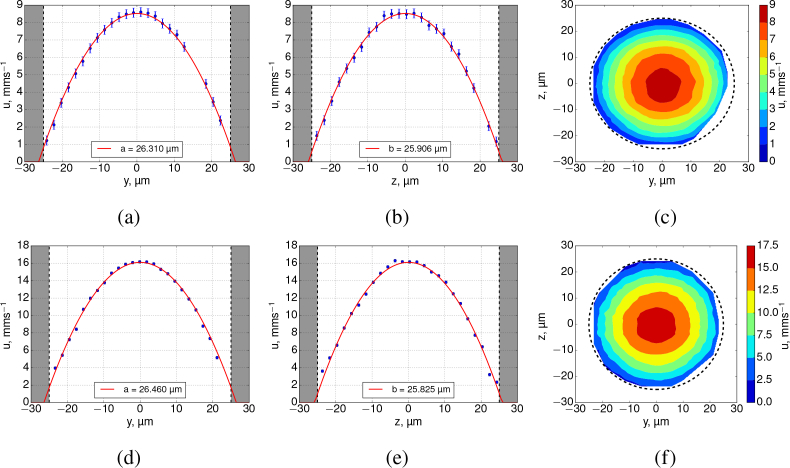
SPIM-μPIV analysis results of FEP tube experiments in locations of peak flow, from analysis of fluorescence imagery. Top row - 0.5 μl/min nominal flow rate beads, bottom row - 1 μl/min beads. Shaded area represents tube walls. The profiles of the *u*-components of velocity in *xy* (a,d) have very similar shapes for all experiments (semi-minor axis ranges ≈26.3 – 26.5 μm). Similarly, the *xz* planes (b,e) show similar profile shape (semi-major axis ranges ≈25.9 – 25.8 μm). This confirms that there is no significant anomalous bias to the flow profile recovered in the *xz* plane, thus validating our use of SPIM-μPIV for 3D flow mapping. (c,f) show colormaps of the velocity magnitude in the *yz* plane, confirming that to a good approximation the reconstructed flow is circularly symmetric and Poiseuille-like. Dashes show a diameter of 50 μm.

For each of the above BF data, the fluorescence equivalent is presented in Fig. 5

. Both *xy* and *xz* planes exhibit a profile shape very close to that of an ideal parabola and do not suffer from severe flattening of the flow profile in the *xz* plane. This is due to the narrow sheet illumination (measured FWHM of approx 2 μm) of the fluorescent beads when using SPIM. The peak values of *u*-velocity components between BF and FLR differed by 8.8% (0.5 μl/min dataset) and 3.9% (for the 1.0 μl/min dataset). Since the illumination depth is the thickness of the light sheet, we assume this to be the DOC of a SPIM-μPIV set-up. Fitted peak velocities, semi-major and minor axes are summarized in Table 2

**Table 2:** Data fitting parameters summarized (see Figure 4 and Figure 5 for data points). The tube radius was measured from the images to be 26.450 ± 0.645 μm. Parameters *a* and *b* are the semi-minor and major axes of a parabolic flow profile. For a parabolic flow in a tube, which drops off to zero at the tube walls, *a* and *b* will match the radius of the tube. Note the dramatic and unphysical variation in the *b* parameter for brightfield datasets. The incorrect profile recovered with BF PIV analysis can be seen in Figure 4.

Data	Flow rate, μl/min	Fitted *V*_max_, mm/s	Fit *a*, μm	Fit *b*, μm
BF	0.5	7.769 ± 0.003	25.591 ± 0.008	39.739 ± 0.033
FLR	“	8.521 ± 0.002	26.310 ± 0.005	25.906 ± 0.004
BF	1.0	15.479 ± 0.011	25.002 ± 0.014	61.565 ± 0.090
FLR	“	16.102 ± 0.004	26.460 ± 0.006	25.825 ± 0.005

.

**Fig. 6:**
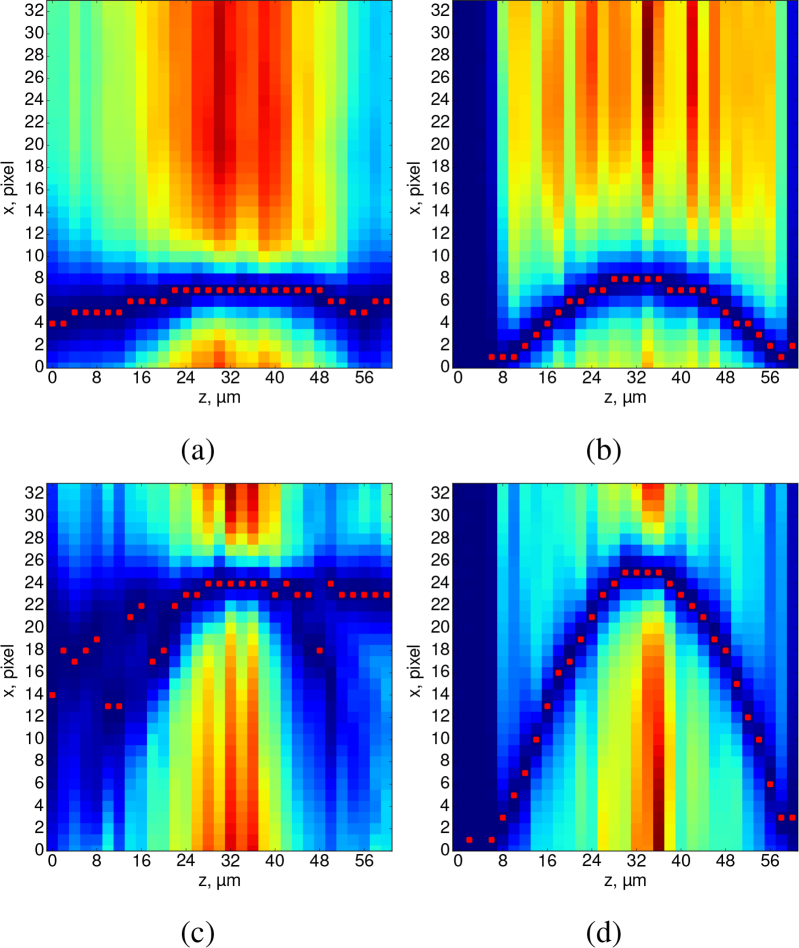
Heatmap visualization of correlation matrices from a *z* stack (representing a single IW selected from our FEP tube data, Figs. 4 and 5), flow rates of 0.5 μl/min (a,b) and 1 μl/min (c,d). These plots give insight into the origin of strong biasing effects that compromise brightfield μPIV analysis. The images represent a cut parallel to the *x* axis through the peak of the correlation matrices (correlation amplitude represented as color), for each *z* plane in our dataset. In μPIV analysis, the flow velocity is determined from the location of the peak correlation matrix value in each plane (annotated with red squares). For the fluorescence data (b, d) it can be seen that the correlation matrices consist of a single well-defined peak at each *z*, with the location of that peak following the expected parabolic profile. However for the brightfield data (a, c) it can be seen that there is significant broadening and clutter present in the correlation matrices which compromises the velocity and leads to the incorrect flattened parabolic profiles shown in Fig. 4. Nevertheless, a “ghost” of the true underlying parabolic profile can be discerned in (c), although its weaker amplitude means it is not correctly identified as the calculated flow value.

Our observations of these flattened flow profiles in BF datasets are consistent with the data presented by Poelma *et al.* [18], who measured blood flow in a vessel of a chicken embryo using both RBCs (BF) and fluorescent tracer particles (epifluorescence). They found that, depending on the objective lens used, RBCs imaged in brightfield can lead to a greater flow underestimation than (smaller) fluorescent tracers, but also a flattening of the overall profile as a function of *z*. They argued that the cause for this flattening of flow profile in the *xz* plane was due to a large DOC when using large tracers such as RBCs. However, while some flattening of the expected parabolic flow at the centre of the channel is indeed anticipated for μPIV measurements using conventional epifluorescence imaging, this does not explain why their estimated profile practically remains flat along the whole depth of the channel in their Figure 10B, as it does in our Fig. 4, rather than being more akin to a simple “averaging” of the profile over some depth range, as would be implied by conventional DOC theory.We propose that the cause of the apparent flow profile flattening is a velocity profile in *z* whose *gradient* varies significantly with *z*, combined with a large depth of correlation. This is of course the case for a parabolic flow profile, where large gradients exist near the walls but gradients are low near the center of the tube. In this case, even when focused some distance from the center of the tube, the integrated contribution of all the particles at the center of the tube ends up dominating the correlation matrix, and hence determining the calculated flow value. Figure 6

illustrates this effect by examining a set of cross-correlation matrices from our experimental data analysis. In Fig. 6(c) in particular, we can recognise a dark blue parabolic arc representing high values in the correlation matrix due to the motion of in-focus particles, but we also see other regions of even higher values that can be understood as the accumulated contributions to the correlation matrix of the large number of (out-of-focus) particles near the center of the tube. Because the velocity gradient is small there (i.e. all have similar velocities), all these particles contribute to the same region of the correlation matrix and produce a large but erroneous peak that does not represent a correct estimate of the true velocity in the focal plane. The effect can be understood most clearly by examining Fig. 6(c), where the peak flow resulted in movement of approximately 6 bead diameters between successive images, but the same effect (non-physical biasing towards the peak velocity) can be seen in Fig. 6(a), where the peak flow was around a third of that – and, of course, also in the flow profiles plotted in Fig. 4. What we are describing can be thought of as a generalization of the widely-recognised issue where structure in the static background in BF images can lead to a failure to recover the correct flow unless specific steps are taken to eliminate that background. However, in this case it is other moving flow tracers forming the background, and so there is no obvious post-processing strategy to eliminate them.

Overall, our results show that SPIM-μPIV can successfully recover the flow profile in a tube of circular cross-section, in contrast to BF-μPIV which suffers from significant bias and recovers an incorrect flow profile. This highlights the need for depth-sectioned imaging for μPIV, as well as illustrating the complex effects at play in BF-μPIV that cannot be understood using a simple DOC model.

### 3.2. Out-of-plane motion tolerance

**Fig. 7:**
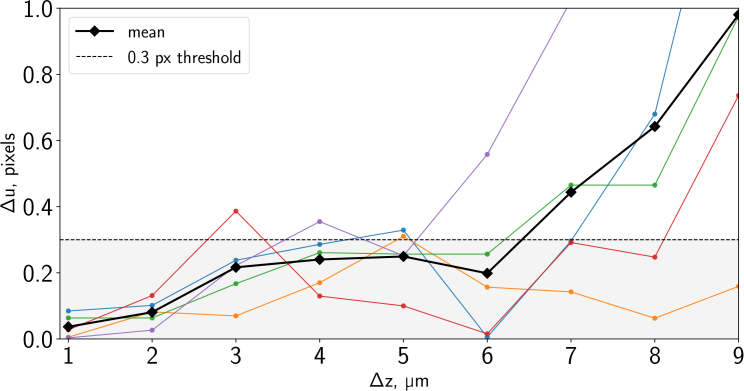
PIV measurement error vs. out-of-plane motion. The 4 solid lines with dots represent individual correlation-averaged results from different volumes in the tube. The bold dashed line with squares indicates the simple mean of the 4 measurements. The finely dashed line marks the acceptable amount of error on the velocity measurement of 0.3 pixels (following [1]). FWHM of the light sheet used in this experiment ≈ 2.5 μm.

Following the procedure described in subsection 2.2, we measured in-plane velocities for datasets acquired using real fluorescent bead samples subject to known out-of-plane motion, and compared the measured velocities to the known synthetic in-plane motion applied. The results are of course subject to statistical variation, and we repeated the experiment four separate times (with independent bead samples). Figure 7

shows the absolute error Δu in our measured *x* velocity, plotted as a function of the known out-of-plane motion Δz. We found that in general our SPIM-μPIV system was able to recover the correct velocity values within less than 0.3px error, for up to 4 μm of out-of-plane motion. Here we chose the rather demanding threshold of 0.3 pixels error based on the convention in engineering PIV applications, where experiments generally use much more regular seeding, and tolerances are tight [1].

This therefore confirms that our approach is appropriate for measuring the two in-plane components of a truly three-dimensional microfluidic flow, as long as the out-of-plane velocity component does not result in motion of more than 4 μm between the two images of a frame pair. We note that one of the major reasons that we can tolerate this level of OOPM is because of our use of correlation averaging to increase the effective number of tracer particles in an IW (both for these validation experiments and for the zebrafish heart flow imaging experiments). This improves the noise characteristics to the point that even a relatively weak correlation peak is still distinguishable above the background noise in the correlation matrix. While clearly an arbitrarily large level of OOPM could not be tolerated (there would be no tracers common to both images of the frame pair), our results illustrate that OOPM of more than the FWHM sheet thickness can be tolerated, if sufficient correlation averaging is applied.

### 3.3. Flow measurements in the zebrafish larva

**Fig. 8:**
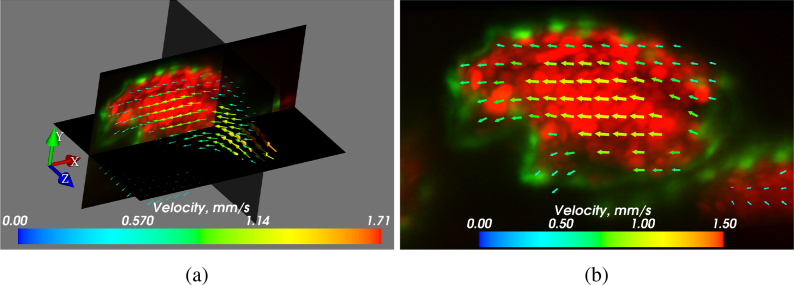
3D flow reconstruction using 2D flow data in the zebrafish heart (walls expressing flk1:GFP and RBCs expressing gata:DsRed). (a) 3D cut-plane image of blood flow in a ∼3 dpf zebrafish heart. Peak flow vectors (corresponding to the high end of the colorbar) are plotted, but not visible in this orientation. For the most part of the heart the calculated flow field is smooth. The erroneous areas mostly correspond to positions where the flow is out-of-plane. The flow measurements are reliable as long as the plane of the flow is reasonably well-aligned with the imaging plane (in-plane motion dominates over out-of-plane motion). The sample mounting in SPIM allows many sample orientation options, however, the geometry of the heart often determines a preferred orientation for flow imaging. (b) Close up of the *xy* plane of (a). Visualization 1 in the supplementary material also shows the flow in the heart, over the full heartbeat. Visualization 2 further illustrates the depth-resolved flow results at a single phase. The square root of velocity magnitudes is displayed to better demonstrate the dynamic range. Visualized using Mayavi [32].

We imaged a ∼3 days post fertilization (dpf) zebrafish larva ( Dataset 2 [26]) according to the procedures described in subsection 2.3. Figure 8

visualizes the flow field in three orthogonal planar cuts through our full 3D dataset, demonstrating our ability to fully resolve the flow as a function of *x*, *y* and *z*.

At this particular age, the valves between the chambers are not yet formed. As a result, the heart does not function as a fully effective pump, and regurgitation of blood in the reverse direction can be observed. The wall shear stress that occurs in the developing heart is considered to be an important stimulus for valve formation [10], and 3D μPIV has the potential to provide precision *in vivo* flow measurements during heart development. Our results in subsection 3.1 have already highlighted the fact that, while flow fields and wall shear have previously been inferred from brightfield imagery (e.g. [10, 27]), quantitative interpretation and analysis of brightfield data should be treated with caution due to the effects of depth of correlation discussed earlier. Our SPIM-μPIV results eliminate this concern due to the depth sectioning provided by the light sheet.

#### 3.3.1. Pumping performance

**Fig. 9:**
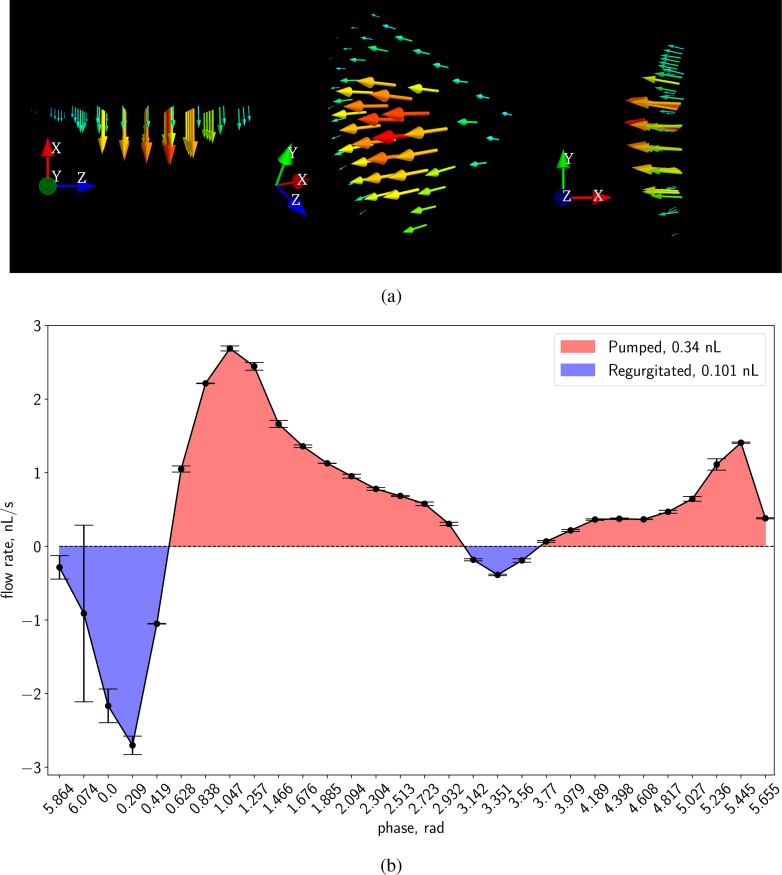
Flow through the atrium. (a) Several orientations of the flow in our chosen cross section through the middle of the atrium at phase bin 1.2 in a ∼3 days old zebrafish atrium. We note that we discarded data well outside the walls of the atrium. Visualized using Mayavi [32]. (b) Net flow rate integrated across this cross section in the atrium, as a function of heart phase. Positive flow (pumping, towards the ventricle) shaded in red; negative flow (regurgitation, out of the atrium and back into sinus venosus) shaded in blue. The area under the curve gives the pumped volume. The plotted error bars were obtained by splitting the data set in two halves (effectively reducing the seeding by half) and using the absolute difference between the flow rate results of two values as the measure of the spread within each phase bin. The least reliable phases correspond to near full contraction of the atrium. Here the flow is complex and bidirectional due to the fact that the valves are not yet fully developed – this, combined with the extremely high flows through the constriction of the partially-formed valve, make flow measurements at this exact location extremely challenging.

As an illustration of the information available from our analysis, we measured the flow rate across a plane located in the atrium (Fig. 9(a)

) throughout the heart cycle, enabling us to quantify this regurgitation in addition to forward pumping. We measured the volume of regurgitated blood (0.101nL per beat), and showed the net pumped volume per beat in the atrium for this fish to be 0.239 nL, with an approximate peak flow rate through the middle of the atrium of ∼ 2.690 nL/s, see Fig. 9(b). These values represent 3D-resolved measurements that serve as refinements to the approximate stroke volumes previously reported in the literature from estimates based on 2D shape projections (e.g. Figure 5A in [28]), as well as single-projection velocity measurements [9, 29].

#### 3.3.2. Phase-resolved correlation averaging

Figure 10

**Fig. 10:**
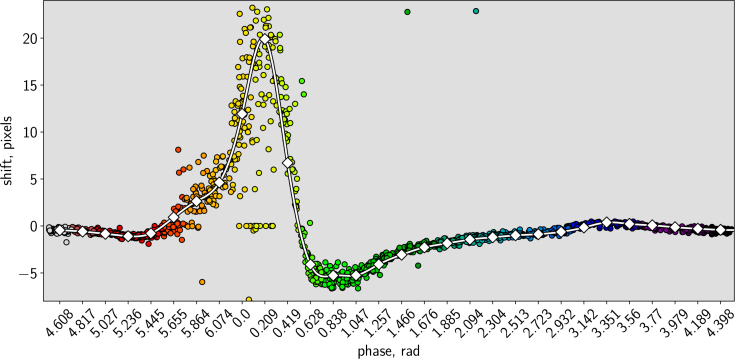
Comparison of correlation averaged (white line with squares) and single-pair (multi-colored dots) *u*-component measurements for 31 phases for interrogation window at (*x* = 239.5px, *y* = 107.5px), which is approximately at the 3/4 of the atrium length from the left edge of the atrium at the 7th *z*-plane (around the middle of the atrium depth-wise). The lack of spread phase-wise suggests that synchronization was sufficiently robust against any beat-to-beat variations in the heart. Note that this plot displays every raw datapoint, with no exclusion of outliers such as frame pairs containing no RBCs. The detailed features of this plot are interpreted further in the main text.

shows a comparison between single-pair and correlation-averaged PIV results, for a single IW located near the centre of the atrium. The effectiveness of the correlation averaging can be seen from the fact that we recover a smooth velocity profile as a function of time, in spite of the significant spread in the raw pixel shifts calculated from individual frame pairs.

The raw and correlation-averaged results around phase 5.6–0.6 deserve further discussion. There is significant spread among the pixel shifts calculated from single frame pairs at phases 5.6–0.2. Although a spread is to be expected in analysis of single frame pairs due to the sparse seeding provided by the RBCs, the spread here is noticeably greater than at other phases. Examination of the raw frame pairs around the region of peak velocity indicates that the increased (vertical) spread in pixel shifts here can be explained by the existence of significant velocity shear within the IW; this suggests that our calculated velocity fields could be further improved in these locations by the use of smaller IWs or more sophisticated PIV analysis algorithms.

In contrast, there is less spread around phases 0.4–0.6, here the velocity is falling rapidly with time. The lack of significant *horizontal* spread here is important evidence of the accuracy with which we are able to assign phases to the individual frame pairs: if there was a significant error associated with our calculated phase values, this would have introduced an artificial spread in phase in the individual datapoints. This lack of spread in phase also vindicates an important implicit assumption in our analysis. In the past, we and other authors have found the motion of the *heart wall* to be highly stereotypical in spite of natural variations in the rhythm of the heartbeat. Indeed, this is an implicit assumption of any synchronized serial acquisition strategy [7, 19, 20], the assumption that there exists an underlying canonical beat sequence that can be sampled stroboscopically across multiple heartbeats. Any blood flow mapping technique aside from a true 3D 3-component snapshot measurement must rely on this same assumption for the flow. However, we are not aware of any previously-published evidence to validate this assumption in small-animal hearts. The lack of horizontal spread noted in Figure 10 is evidence that the *flow*, as well as the wall motion, is indeed highly reproducible between beats, thus confirming that correlation averaging and sequential planar imaging is applicable.

#### 3.3.3. Limitations

In our method and results presented here, we have resolved velocity vectors as a function of position (*xy* resolution determined by interrogation window size; *z* resolution determined by light sheet thickness), but our analysis only quantifies the two in-plane components of the three-dimensional velocity vectors at each point in space, i.e. only 2 of the 3 velocity components are measured.

As discussed earlier, out-of-plane motion can cause a reduction in the signal peak. We carefully oriented the zebrafish so that the inflow and outflow from the atrium were approximately in-plane. This minimizes (although of course does not fully eliminate) OOPM, ensuring that the OOPM remains within acceptable limits (see previous section). We have therefore made a rough estimate of the maximum OOPM in our actual dataset, by approximating the heart chamber as a symmetrically-contracting cylinder and searching for the largest *v*-component (the component which is parallel to the vertical *y*-axis) throughout the volume of the heart. Under this assumption of cylindrical symmetry, this component also serves as an estimate of the maximum out-of-plane motion we expect to be present within the volume of the heart.

We investigated the region of the atrium (horizontal coordinates from 120 to 320 pixels in the raw data), which contains 176 interrogation windows used in PIV analysis. At most, for any one depth-plane and phase, we observed 6 IWs which measured *v*-component values above 4 μm threshold, which we found experimentally for fluorescent beads in subsection 3.2. We note that most of these values are at the atrium and sinus venosus (SV) junction, and SV is at about 45 degrees to the atrium in the current sample orientation, therefore the larger *v*-component values are not unexpected here. These therefore probably represent a significant overestimate of the actual out-of-plane motion present in our data.

We note that it is possible to minimize the impact of out-of-plane motion for a frame pair by choosing a sufficiently small inter-pulse time between two exposures [17, p.176], or by using correlation averaging as we have demonstrated empirically in Fig. 7.

In future we intend to further quantify the performance of our SPIM-μPIV in the presence of OOPM in the real biological environment of the circulatory system, as a function of the level of OOPM and the level of correlation averaging that is applied. Although we are currently limited to measuring the two in-plane components of the velocity, in future we expect it will be possible to measure all three velocity components (3C-3D μPIV) by fusion of information acquired from different viewpoints, either by sequential imaging of the sample in different orientations, or by simultaneous multi-view imaging (e.g. [30]).

## 4. Conclusions

We have demonstrated for the first time, to our knowledge, that robust, correctly *xyz*-resolved (3D-2C) μPIV can be achieved by using light sheet microscopy (SPIM) to provide depth sectioning. This eliminates complex issues arising in brightfield and epifluorescence μPIV, associated with depth-of-correlation effects. Indeed, we have shown that these depth-related issues in volume-illumination approaches have the potential to be even more severe than conventional depth-of-correlation theory would indicate.

Our validation using a tube phantom confirms that our approach permits μPIV measurements with ∼2 μm *z* resolution, showing its potential for a wide range of applications in microfluidics. Our light sheet-based approach is especially valuable when imaging living organisms, such as the zebrafish, where photobleaching and phototoxicity are important considerations. For *in vivo* applications where seeding densities are often insufficient for high quality 3D flow reconstructions, we have demonstrated the use of heart synchronisation techniques in conjunction with correlation averaging to improve the statistical quality of the results by combining information from multiple heartbeats.

Our approach directly permits 3D resolving of the two in-plane velocity components (2C-3D resolution). This is sufficient for many applications such as flow volume and wall shear stress measurements, especially since the sample orientation can easily be controlled in SPIM imaging. While care must be taken when interpreting the full volume results, as only 2-component vectors are captured, our work is an improvement over previous work that has used data taken at only one focal plane in the heart. We note that, if desired, our approach has the potential to extend to full 3C-3D flow imaging through the use of stereoscopic PIV for example. With time-resolved *in vivo* studies of function, development and disease in animal models such as the zebrafish becoming increasingly common, we have shown how robust, high-resolution flow fields can be recovered from inside the complex environment of a living organism.
